# Cost-effectiveness analysis of surgical versus non-surgical management of acute Achilles tendon ruptures

**DOI:** 10.1007/s00167-018-4953-z

**Published:** 2018-04-25

**Authors:** Olof Westin, Mikael Svensson, Katarina Nilsson Helander, Kristian Samuelsson, Karin Grävare Silbernagel, Nicklas Olsson, Jón Karlsson, Elisabeth Hansson Olofsson

**Affiliations:** 1 Department of Orthopaedics, Institute of Clinical Science at Sahlgrenska Academy, University of Gothenburg, Gothenburg, Sweden; 2 Sahlgrenska University Hospital, Mölndal, Sweden; 3 Department of Physical Therapy, University of Delaware, Newark, DE USA; 4 Health Metrics, The Sahlgrenska Academy, University of Gothenburg, Gothenburg, Sweden; 5 Institute of Health and Care Sciences, Sahlgrenska Academy, Gothenburg, Sweden

**Keywords:** Achilles tendon rupture, HRQoL, ICER, QALY, Sensitivity analyses, Surgical, Non-surgical, Treatment

## Abstract

**Purpose:**

An Achilles tendon rupture is a common injury that typically affects people in the middle of their working lives. The injury has a negative impact in terms of both morbidity for the individual and the risk of substantial sick leave. The aim of this study was to investigate the cost-effectiveness of surgical compared with non-surgical management in patients with an acute Achilles tendon rupture.

**Methods:**

One hundred patients (86 men, 14 women; mean age, 40 years) with an acute Achilles tendon rupture were randomised (1:1) to either surgical treatment or non-surgical treatment, both with an accelerated rehabilitation protocol (surgical *n* = 49, non-surgical *n* = 51). One of the surgical patients was excluded due to a partial re-rupture and five surgical patients were lost to the 1-year economic follow-up. One patient was excluded due to incorrect inclusion and one was lost to the 1-year follow-up in the non-surgical group. The cost was divided into direct and indirect costs. The direct cost is the actual cost of health care, whereas the indirect cost is the production loss related to the impact of the patient’s injury in terms of lost ability to work. The health benefits were assessed using quality-adjusted life years (QALYs). Sampling uncertainty was assessed by means of non-parametric boot-strapping.

**Results:**

Pre-injury, the groups were comparable in terms of demographic data and health-related quality of life (HRQoL). The mean cost of surgical management was €7332 compared with €6008 for non-surgical management (*p* = 0.024). The mean number of QALYs during the 1-year time period was 0.89 and 0.86 in the surgical and non-surgical groups respectively. The (incremental) cost-effectiveness ratio was €45,855. Based on bootstrapping, the cost-effectiveness acceptability curve shows that the surgical treatment is 57% likely to be cost-effective at a threshold value of €50,000 per QALY.

**Conclusions:**

Surgical treatment was more expensive compared with non-surgical management. The cost-effectiveness results give a weak support (57% likelihood) for the surgical treatment to be cost-effective at a willingness to pay per QALY threshold of €50,000. This is support for surgical treatment; however, additionally cost-effectiveness studies alongside RCTs are important to clarify which treatment option is preferred from a cost-effectiveness perspective.

**Level of evidence:**

I.

## Introduction

Achilles tendon rupture is a common injury, which frequently affects middle-aged individuals in the middle of their most productive years [8, 10, 12]. It typically affects patients between the ages of 35 and 45 years with a moderate to high level of activity [8]. It is one of the most common tendons to rupture, with an annual incidence of 13-55/100,000, and is 5–10 times more common among men [8, 10, 12]. The annual incidence of Achilles tendon ruptures has increased significantly in the last 20 years, resulting in increased health-care costs. As treatments become more advanced, health-care costs continue to increase, which places greater demands on health professionals to prioritise cost-effective treatments [1, 15]. In recent years, increased pressure has been placed on physicians to consider the most cost-effective treatment plans and, moreover, to consider the overall impact on society in terms of sick leave and quality of life.

Two main approaches are available for Achilles tendon rupture treatment, both of which yield good clinical outcomes [6, 22, 25]. However, there is still an ongoing debate about the preferred treatment approach. The two main treatment options available are surgical repair of the tendon, using either an open or a percutaneous technique, and non-surgical treatment, using a functional brace. Several randomised, controlled trials (RCTs) and meta-analyses have compared the differences in clinical outcome between the two treatment alternatives [6, 14, 16, 24, 25]. A recent meta-analysis has reported that the incidence of re-ruptures is lower with the surgical technique (3.7% compared with 9.8% using a non-surgical approach) and that there is a greater improvement in early function and a quicker return to work [6]. However, well-known complications, such as wound infections, skin necrosis and injury to the sural nerve, are associated with surgery. Traditionally, the surgical options have been the method of choice for athletes and younger patients with high functional demands [11]. Elderly patients and patients with comorbidities, such as diabetes, have typically been treated non-surgically [11]. There is still no consistent evidence that one treatment is superior to the other. Recent evidence suggest that a decline in surgical management in favour for non-surgical management [19, 20].

A previous study has evaluated the cost-effectiveness of open Achilles tendon repair compared with non-surgical management [7]. That study showed that percutaneous and non-surgical management provided a significant cost reduction compared with open surgery. Moreover, a study by Carmont et al. [4] compared open with percutaneous surgery. These researchers found that percutaneous surgery resulted in lower direct costs with comparable clinical outcomes. However, they reported surprisingly long hospital stays and the indirect costs were not investigated. A recent retrospective cost-minimisation analysis by Truntzer et al. [23], comprising more than 5000 patients, concluded that the cost of non-surgical management was significantly lower than that of surgical treatment. However, to the best of our knowledge, there is no study that compares the cost of open surgical repair and non-surgical management using early weight-bearing and a functional brace in a prospective RCT.

The purpose of this study was to address this knowledge gap and perform a cost-effectiveness analysis comparing surgical with non-surgical management in patients with an Achilles tendon rupture. The secondary aim was to analyse the duration of sick leave between the two treatment groups.

## Materials and methods

Data for this study were collected prospectively alongside an RCT. All the patients that were part of the 1-year follow-up were included. Unfortunately, the re-ruptures (*n* = 5) in the non-surgical group did not complete the 1-year EQ-5D assessments, but a sensitivity analysis in which their costs are calculated and included was performed. The economic analyses were performed by a professor in health economics.

### The randomised, controlled trial

A single-centre randomised, controlled trial was performed at Sahlgrenska University Hospital in Sweden between April 2009 and October 2010 [16]. A total of 201 patients presented with an acute total Achilles tendon rupture during this time period. All the patients had sustained a closed mid-substance Achilles tendon rupture. The diagnosis was made by clinical examination (positive Thompson test, a palpable substance defect) and medical history. The exclusion criteria were ruptures presenting more than 4 days after the initial injury, diseases affecting lower-limb function, such as any neuromuscular disease, diabetes, peripheral vascular disease, immunosuppressive therapy including corticosteriods, skin or wound infection, and inability to attend rehabilitation or follow-up evaluations. All the participants were informed of the study before taking part in randomisation. Ethical approval was obtained from the regional ethical review board in Sweden, DNR 032-09.

**Fig. 1:**
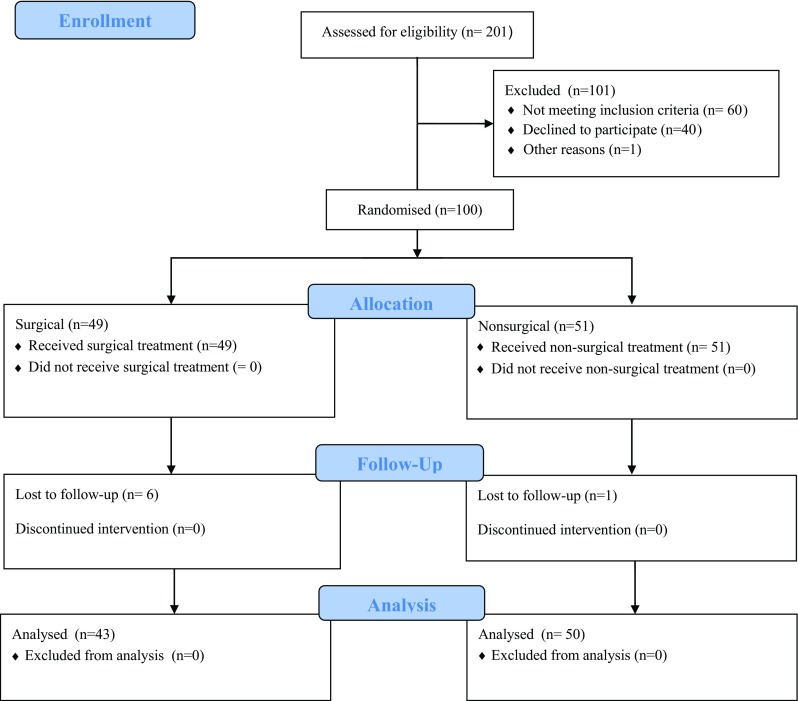
Flow diagram of the study

One hundred patients were included and their median age was 40 (18–65 years). Randomisation was performed directly after inclusion and computer-generated opaque, sealed envelopes were used. One of the surgical patients was excluded due to a partial re-rupture and five surgical patients were lost to the 1-year follow-up (Fig. 1). One patient was excluded due to incorrect inclusion and one was lost to the 1-year follow-up in the non-surgical group (Fig. 1).

### Treatment method

#### Surgical treatment

Forty-nine patients were randomised to surgical treatment. Ten experienced orthopaedic surgeons performed a standardised operation. All the procedures were performed under local anaesthesia and prophylactic antibiotics (cloxacillin) were administered. Because of the high risk of deep venous thrombosis (DVT), prophylactic dalteparin was administered to all patients. Patients were operated on in a prone position, without a tourniquet. Through a postero-medial incision, the paratendon was divided. The rupture site was identified and repaired using end-to-end core sutures with two strong, semi-absorbable sutures (No. 2 Orthocord, DePuy Mitek, Norwood, Massachusetts, USA), using a modified Kessler technique. A running circumferential suture with absorbable sutures (No. 0 Polysorb, Tyco, Norwalk, Connecticut, USA) was used, with an epitendinous cross-stitch technique described by Silfverskiold and Andersson to reinforce the core suture [21].

The foot was placed post-operatively in plantar flexion. A maximum of 20° of plantar flexion was used to fit in the brace. The paratendon was closed with absorbable sutures. The skin was closed with interrupted nylon sutures. Post-operatively, the ankle was placed in a pneumatic walker brace (Aircast XP Diabetic Walker, DJO Global, Carlsbad, California, USA) including three heel pads to create an angle of 22°. Patients were allowed full weight-bearing in this functional brace from the first post-operative day.

All patients were treated with a brace for 6 weeks. Three experienced physiotherapists supervised the post-operative care and standardised treatment was used 2 weeks post-operatively [16]. Rehabilitation protocols have been published elsewhere and are available online [16].

#### Non-surgical treatment

The non-surgical group consisted of 51 patients. Their treatment was initiated immediately after randomisation using the same functional brace as the surgical group, including three heel pads. Full weight-bearing was encouraged from day 1 in a manner similar to the surgical group. This group used a slightly different standardised protocol and was treated with a brace for 8 weeks. Three experienced physiotherapists supervised the rehabilitation and protocols are available online [16].

### Patient outcomes

#### Quality-adjusted life years

Quality-adjusted life years is a measurement that combines health-related quality of life and life expectancy in one metric. Quality of life is measured on an index that is anchored so that 1 represents the best possible health state and 0 represents “equal to being dead”, while life expectancy is measured in years. In terms of interpretation, one QALY is equivalent to living 1 year in the best possible health state. In health-economic evaluation methods (cost-effectiveness/utility analyses), QALY is the most commonly used outcome metric. It was intended to be used for economic analysis where the cost per QALY could be assessed and compared across treatments [3].

Life expectancy is not affected by the surgical or non-surgical treatment, so any difference in the number of gained QALYs is due here to differences in health-related quality of life “QALY weight”. The “QALY weight” was assessed using the EuroQol-5 Dimension Questionnaire (EQ-5D) instrument, which is a generic instrument where patients self-report their health status based on five dimensions (mobility, self-care, usual activities, pain/discomfort and depression/anxiety), including answers on three levels (none, moderate and severe problems). The EQ-5D answers were scored on the index scale based on the UK tariff, with a range of − 0.59 to 1 (Dolan algorithm). Brazier et al. evaluated the EQ-5D in a group of patients with osteoarthritis of the knee and concluded that it could be used for economic evaluations of surgery [2].

Quality-adjusted life years calculations were made at patient level, reflecting the change from baseline, 3, 6 and 12 months.

### Economic costs

The economic costs were categorised as either direct health-care costs or indirect (productivity) costs. The direct costs include resource use for administration, staff salaries and accommodation at recovery. Moreover, they include patient-specific expenses such as examination, surgery (operating room (OR) including anaesthesia and material), post-operative visits, rehabilitation, laboratory tests, or imaging. All costs were collected from the hospitals’ accounting databases. See Table 1 for the list of resource use items and the associated unit costs.

**Table 1:** Resource use units and cost per unit

Item	Cost (EUROs)
Accident & emergency department visit	209
Physiotherapy visit	62
Inpatient night	536
Day surgical bed	267
Surgeon cost per min	5.6
Operation cost per min	16.2
Outpatient clinic visit	185
Magnetic resonance imaging scan	399
Ultrasound scan	267
Prescription drugs	75

Productivity loss was based on the number of sick leave days. The human capital method was used to assess the value of production loss due to sick leave, which implies that each hour of production loss is valued by the gross wage, including social fees (i.e. the market price in the sense that this is what the employer pays per hour). Data on the number of sick leave days were self-reported at the follow-up.

Costs are presented in Euros using 2013 exchange rates for conversion from Swedish kronor (8.86 SEK = 1 Euro).

### Cost-effectiveness analysis

The cost-effectiveness of the surgical treatment was compared with that of the non-surgical treatment based on the incremental cost-effectiveness ratio (ICER) from a societal perspective (including productivity effects). The ICER is calculated as:

$${\text{ICER}}=\frac{{{\text{Cos}}{{\text{t}}_{{\text{surgical}}}} - {\text{Cos}}{{\text{t}}_{{\text{non-surgical}}}}}}{{{\text{QALY}}{{\text{s}}_{{\text{surgical}}}} - {\text{QAL}}{{\text{Y}}_{{\text{non-surgical}}}}}}.$$

The ICER can be interpreted as the cost of obtaining one extra QALY and enables comparisons between interventions in all areas of health care [5]. We did not discount health benefits or costs, as we used a 12-month time horizon.

### Assessing uncertainties

**Fig. 2:**
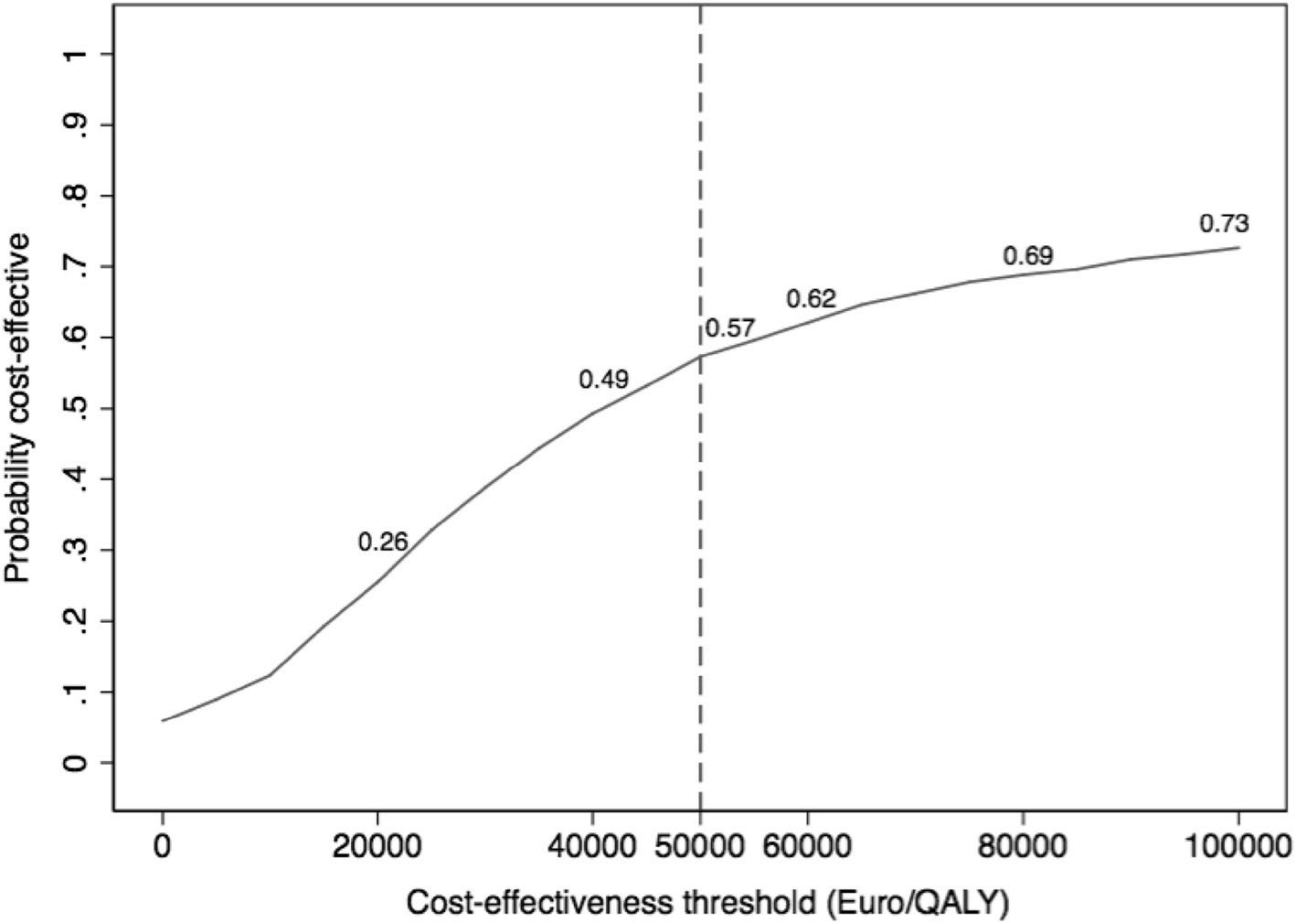
Cost-effectiveness acceptability curve

To demonstrate the sampling uncertainty that surrounds the mean ICER, a non-parametric bootstrapping (with replacement) was conducted. The results of the bootstrapping are shown using a cost-effectiveness acceptability curve (Fig. 2).

The cost-effectiveness acceptability curve (CEAC) is used to demonstrate how many of the bootstrapped ICERs are cost-effective at a given value for what the payer is willing to pay per QALY. The CEAC thus shows the likelihood that surgical treatment is cost-effective compared with non-surgical treatment at different willingness-to-pay thresholds. In the interpretation of results, the maximum willingness to pay was set at a level of 50,000 EUR (443,000 SEK), based on the Swedish National Board of Health and Welfare. (https://www.socialstyrelsen.se/SiteCollectionDocuments/metodbeskrivning-nationella-riktlinjer.pdf).

### Statistical analysis

The data analysis was performed using Statistical analysis system (SAS/STAT, version 14.2, 2016; SAS Institute Inc., Cary, North Carolina, USA). Summary statistics are given in terms of means and standard deviations (continuous variables) and proportions (dichotomous variables). Tests of differences in means were conducted by *t* tests (continuous variables) and equality of proportions using large-sample statistics. It is well known that health-care cost data are typically not normally distributed (right-skewed) and we, therefore, performed sensitivity tests based on logarithmetic transformations.

## Results

Table 2 shows the demographics and economic costs for the two treatment alternatives.

**Table 2:** Summary statistics on demographics and clinical variables of interest

	Total (*n* = 93)	Surgical (*n* = 43)	Non-surgical (*n* = 50)	*p* value
Gender				
Male	80 (86.0%)	34 (79.1%)	46 (92.0%)	
Female	13 (14.0%)	9 (20.9%)	4 (8.0%)	n.s
Age	39.3 (9.2)	38.9 (8.7)	39.7 (9.7)	n.s
Income (EUROs/month)	3711 (1563)	3505 (1560)	3887 (1561)	n.s
Re-rupture				
Yes	6 (6.5%)	1 (2.3%)	5 (10.0%)	
No	87 (93.5%)	42 (97.7%)	45 (90.0%)	n.s
Hospital admission				
Yes	5 (5.4%)	3 (7.0%)	2 (3.8%)	
No	88 (94.6%)	40 (93.0%)	51 (96.2%)	n.s
# Doctor visits	4.31 (1.62)	4.79 (1.15)	3.90 (1.85)	n,s
# Physiotherapy visits	26.6 (13.2)	28.2 (13.1)	25.3 (13.3)	n.s
# Sick days	21.2 (25.5)	17.8 (19.0)	24.1 (29.9)	n,s
Direct cost	3869 (1 704)	5007 (1 009)	2890 (1 571)	< 0.001
Indirect cost	3073 (3 833)	2675 (3 365)	3416 (4 198)	n.s
Total cost	6942 (4 116)	7682 (3 621)	6305 (4 435)	n.s

All costs are presented in EURO

For categorical variables *n* (%) is presented and for continuous variables mean (standard deviation). *p* values based on the null hypothesis of equal proportions (dichotomous variables) and means (continuous variables) using large-sample equal proportions test and *t* tests, respectively

There were no significant differences in terms of age, side or gender between the two treatment groups. The non-surgical group had five re-ruptures and the surgical group had a partial re-rupture. The mean differences in physician and physiotherapist visits were not statistically significant. Surgical management demonstrated shorter sick leave with a mean(SD) of 17.8 (19) days compared with 24.1 (29.9) days for non-surgical management, but, given the large variation, this difference was not statistically significant. The total cost of surgical treatment was higher (7682 versus 6305 EUR, *p* = 0.024) and was statistically significant both using a t-test assuming a normal distribution and when comparing cost differences that were log-transformed. The higher costs of surgical treatment are explained by higher direct costs, whereas the indirect costs are lower, due to fewer days of sick leave.

All five re-ruptures subsequently underwent surgical reconstruction. Details on surgical data are shown in Table 3. The average time for re-rupture surgery was  163.4 (13.3) min compared with a primary repair of 87 (22.8) min (*p* < 0.001). The average total surgical cost of a primary repair was 2087 (432) EUR, while it was 3615 (219) for re-rupture surgery.

**Table 3:** Surgical data for patients randomised to surgical treatment and non-surgical patients operated due to re-ruptures

	Total (*n* = 48)	Surgical (*n* = 43)	Re-ruptures in the non-op group (*n* = 5)	*p* value
Op time (min)	94.9 (32.2)	87.0 (22.8)	163.4 (13.3)	< 0.001
Knife to skin time	56.1 (19.5)	52.3 (14.9)	88.6 (26.1)	< 0.001
Surgeon cost	2972 (1035)	2772 (788)	4696 (1384)	< 0.001
Surgical cost	1964 (627)	1805 (432)	3333 (219)	< 0.001
Total surgical cost	2246 (627)	2087 (432)	3615 (219	< 0.001

For categorical variables *n* (%) is presented and for continuous variables mean (standard deviation). *p* values based on the null hypothesis of equal proportions (dichotomous variables) and means (continuous variables) using large-sample equal proportions test and *t* tests, respectively

### Complications

There was one partial re-rupture in the surgical group. The non-surgical group had five re-ruptures (9.6%). In the surgical group, six (12.5%) patients had a superficial wound infection treated with oral antibiotics. Deep vein thrombosis occurred in one patient (2%) in the surgical group and two patients (3.8%) in the non-surgical group. One patient in the surgical group suffered a sural nerve injury.

Table 4 demonstrates how the groups compared in terms of health-related quality of life and QALY estimates. The groups were similar at baseline. At 3, 6 and 12 months, the sample mean EQ-5D scores were slightly higher in the surgical group, but this was not statistically significant (n.s.) The QALYs are calculated as the “area under the curve”, i.e. taking account of the time spent in each health state. The mean QALYs are 0.89 and 0.86 in the surgical and non-surgical group, respectively.

**Table 4:** Health outcomes based on EQ-5D scores and QALY estimates

	Total (*n* = 88)	Op (*n* = 43)	Non-op (*n* = 45)	*p* value
EQ-5D baseline	0.962 (0.105)	0.976 (0.069)	0.948 (0.130)	n.s
EQ-5D 3 months	0.755 (0.152)	0.788 (0.163)	0.723 (0.135)	n.s
EQ-5D 6 months	0.875 (0.155)	0.892 (0.174)	0.859 (0.136)	n.s
EQ-5D 12 months	0.904	0.913	0.894	n.s
Mean QALYs (95% CI)	0.88 (0.85–0.90)	0.89 (0.85–0.94)	0.86 (0.83–0.89)	n.s

Mean EQ-5D score shown at each point of measurement (standard deviation) and mean QALYs (95% CI)

The cost-effectiveness results are shown in Table 5 and once again indicate that surgical treatment is more expensive but is also associated with a slightly better health outcome. Dividing the difference in costs by the difference in QALYs gives a cost per QALY of 45,855 EUR. We do not report the confidence intervals of the cost-effectiveness ratio, as it is not well defined (includes negative ratios). Instead, the variability in the results is assessed in more detail below.

**Table 5:** Cost-effectiveness results

	Difference in QALY (95% CI)	Difference in cost (95% CI)	ICER
Surgical versus non-surgical	0.03 (− 0.02 to 0.08)	1377 Euros (− 308 to 3062)	45,855 Euro/QALY

### Sampling uncertainty

There is considerable uncertainty in the assessed cost-effectiveness ratio, especially considering the relatively small difference in health benefits. A non-parametric bootstrapping with replacement to assess the sampling uncertainty (1000 bootstrap replicates) was, therefore, conducted. The results are presented in a cost-effectiveness acceptability curve, which plots the probability that the surgical treatment is cost-effective for a range of “threshold values” for the decision makers’ willingness to pay per QALY (Fig. 2). At a willingness to pay per QALY of 50,000 Euros, there is a 57% likelihood that surgical treatment is cost-effective compared to the non-surgical treatment, which increases to 69% at a threshold value of 80,000 Euros and 73% at a threshold value of 100,000 Euros.

## Discussion

The results show that the cost per QALY of surgical treatment versus non-surgical treatment for patients with Achilles tendon ruptures was 45,855 Euros. This can be compared with a “rule-of-thumb” value for determining moderate cost-effective treatments by the Swedish National Board of Health and Welfare at approx. 50,000 Euros, where it is 57% likely that surgical treatment is cost-effective. The cost of surgical management was higher, due to costs that are associated with the surgical procedure per se. The mean cost of the surgical procedure for a primary repair in this study was 1805 (432) Euros. However, the surgical group has a slighty higher EQ-5D at 3, 6 and 12 months. These differences were, however, not statistically significant. From a societal perspective, there were no statistically significant differences between the groups. Average salaries between the groups were comparable.

### Total costs

#### Direct costs

With increasing health-care costs, there is an increasing need to consider the financial and economic consequences of health-care treatment. This may be especially true in orthopaedics, where there is often a choice between surgical and non-surgical treatment [1, 9, 23]. In a previous study by Carmont et al. [4], the direct costs were lower for percutaneous surgery compared with open surgery. A recent cost-minimisation analysis by Truntzer et al. [23] concluded that non-surgical management is cost-effective due to the difference in direct costs. However, they did not assess any functional outcome data and did not, therefore, consider the patient benefits.

#### Indirect costs (production loss)

In this study, the mean number of days of sick leave was higher in the non-surgical group, which is in line with previous data from Möller et al. [13]. This is due in part to the five re-ruptures in the non-surgical group, which had a mean sick leave period of 26.8 (12) days. Studies that do not take the indirect costs into consideration thus overestimate the cost difference by not accounting for the negative economic consequences for society and patients due to sick leave. There is a large variation in production loss between different patients; some have no time off work, while others are off for 3 months. This is probaby due to the difference in the type of occupation, as well as the attitude of the individual patient. With physically demanding jobs, it is difficult/impossible to return to work until the tendon is completely rehabilitated, whereas a desk job would not require any work loss. Insurance issues may also play a part, as some employers will not let their employees work while wearing a boot. However, this plays little part in this study, as there is no such restriction in Sweden. However, this should be considered if extrapolating or transferring results to other health-care insurance contexts.

#### Quality-adjusted life years

No previous study has used QALY to evaluate the cost-effectiveness of surgical versus non-surgical treatment, which is important to compare the results with other types of intervention in the health-care sector. It should be noted that the five re-ruptures were excluded from the economic evaluation due to the fact that they were lost to the EQ-5D follow-up. It can be assumed from previous studies that they would obtain poorer scores than the average non-surgical patient [17, 18] and the cost per QALY difference could, therefore, be greater than demonstrated.

### Surgical versus non-surgical management

A recent systematic review by Deng et al. [6] assessing eight RCTs involving a total of 762 patients found that the risk of re-rupture was significantly higher with non-surgical treatment. No differences were found in functional outcome. This result is in line with the present study, with five re-ruptures in the non-surgical group and only one partial re-rupture in the surgical group. No significant differences were seen in the Achilles tendon total rupture score at 1 year [16]. Patients who sustain a re-rupture will subsequently undergo surgical treatment that is more complicated, at an average cost of 3333 Euros, and studies suggest that these patients have a worse long-term outcome [17, 18]. The incidence of patients treated surgically has recently been shown to be declining [19]. It could potentially be beneficial to operate on more patients to avoid re-ruptures and the cost associated with them. This could lead to lower costs associated with this group and could be beneficial from a cost-effectiveness perspective.

The main strength of this study is its prospective nature and the fact that the data were collected alongside a randomised controlled trial. All the data are patient reported. One limitation is that the costs are calculated from the Swedish perspective, which implies that the results may not be directly transferable to other countries and different health-care systems. Another limitation is that we were not able to include the re-ruptures in the health-related quality-of-life follow-up due to the fact that they were excluded from the 1-year follow-up in the original RCT.

The results of the present study provide us with further knowledge in the never-ending debate on the optimal treatment after an Achilles tendon rupture. This is a much-debated topic, where many different factors play a role, and this economic evaluation indicated that, at a cost-effectiveness threshold of 50,000 Euros, surgical management could be considered cost-effective. One factor that needs to be considered is patient choice. With the easy accessible information that today’s society provides, patients often express their own opinion on whether or not to undergo surgery. This can play a crucial role in their motivation for rehabilitation and return to work and might, therefore, influence the cost.

## Conclusion

Surgical management was more expensive compared with non-surgical management. However, the cost-effectiveness results indicate that surgical treatment is 57% likely to be cost-effective if the willingness to pay per QALY is €50,000. This is support for surgical treatment, however, additionally cost-effectiveness studies alongside RCTs are important to clarify which treatment option is to prefer from a cost-effectiveness perspective.
